# Traumatic Open Anterior Hip Dislocation in an Adult Male: A Case Report

**DOI:** 10.7759/cureus.2862

**Published:** 2018-06-22

**Authors:** Anderson Freitas, Vincenzo Giordano Neto, Patrick F Godinho, Silvio L Macedo, Vinicius F Ribeiro de Oliveira, Gary Alan A Montano, Celio Silva, Vanessa C Bandeira

**Affiliations:** 1 Instituto De Pesquisa E Ensino-Ipe, Hospital Ortopedico E Medicina Especializada - Home, Brasilia, BRA; 2 Orthopedics, Hospital Municipal Miguel Couto, RIo De Janeiro, BRA; 3 Instituto De Pesquisa E Ensino-Ipe, Hospital Ortopedico E Medicina Especializada-Home, Brasilia, BRA; 4 Instituto De Pesquisa E Ensino-Ipe, Hospital Ortopedico E Medicina Especializada - Home, Braslia, BRA

**Keywords:** anterior hip dislocation, open dislocation of hip, trauma, femoral head necrosis

## Abstract

This case report describes the surgical treatment and one-year follow-up of an adult male patient, who was treated for a severe anterior open hip dislocation fracture with no sign of femoral head necrosis and maintaining a Harris Hip Score (HHS) of 93.

## Introduction

The hip is a joint that has great stability, intrinsically due to its ball-and-socket shape and extrinsically due to its ligaments and strong muscles. A high-energy trauma is needed to cause the dislocation of the hip. Anterior hip dislocations are very uncommon and constitute 10%-15% of the traumatic displacements of this joint. However, when they do occur, they could be associated with brain, thoracic, and abdominal lesions as well as local lesions, such as neurological or severe vascular injuries, requiring immediate intervention. A reserved prognosis, due to the high rate of complications such as deep infections, femoral head necrosis, severe functional limitation, and arthrosis, is observed in several published case reports [1- 5]. The authors describe the follow-up of a fracture and a severe anterior dislocation of the hip in a young adult who surprisingly had a good outcome in the one-year follow-up, with a Harris Hip Score (HHS) of 93, no necrosis of the femoral head, and with only a slight degree of arthrosis.

## Case presentation

**Figure 1:**
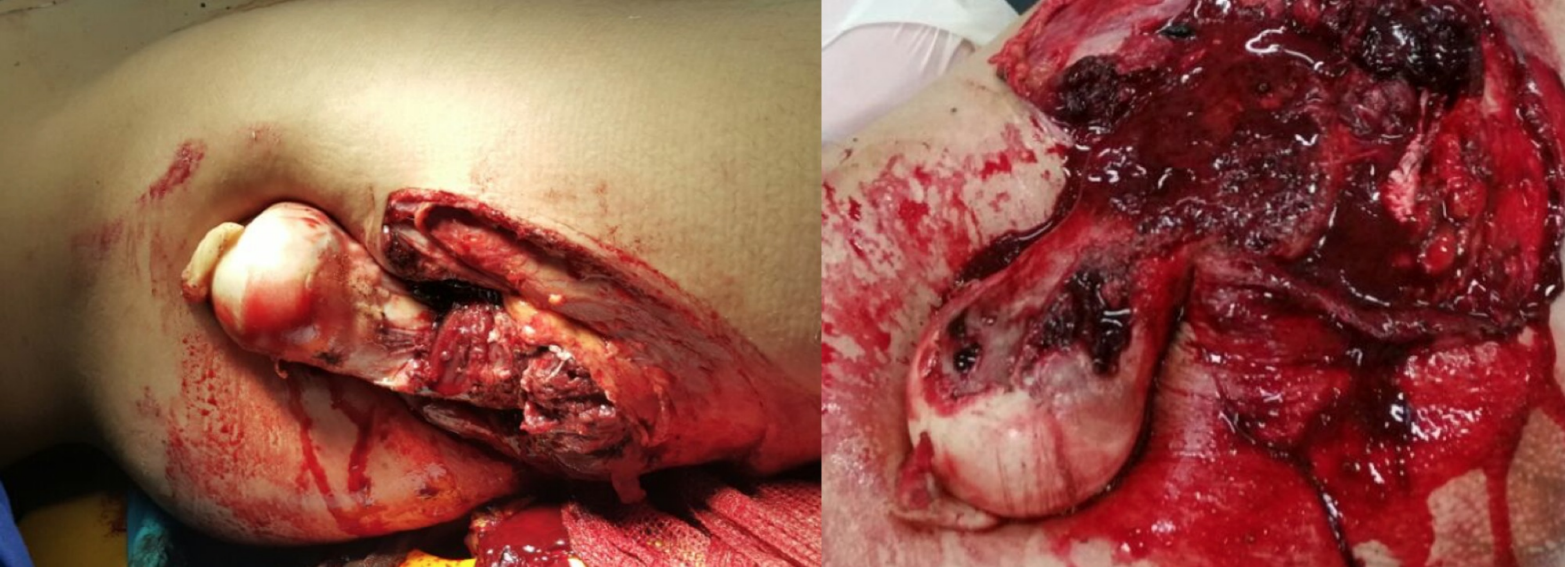
A photograph of the lesion in the right hip region, showing the exposure of the entire proximal end of the femur

We present the case of a 28-year-old male who suffered a high-energy motorcycle accident. At admission, the patient was conscious, Glasgow coma scale (GCS) 15, hemodynamically stable, and presenting superficial excoriations on the trunk and lower limbs. However, there was a wound of approximately 20 cm on the lateral aspect of the right hip at the level of the greater trochanter, exposing the entire proximal end of the femur (Figure 1).

**Figure 2:**
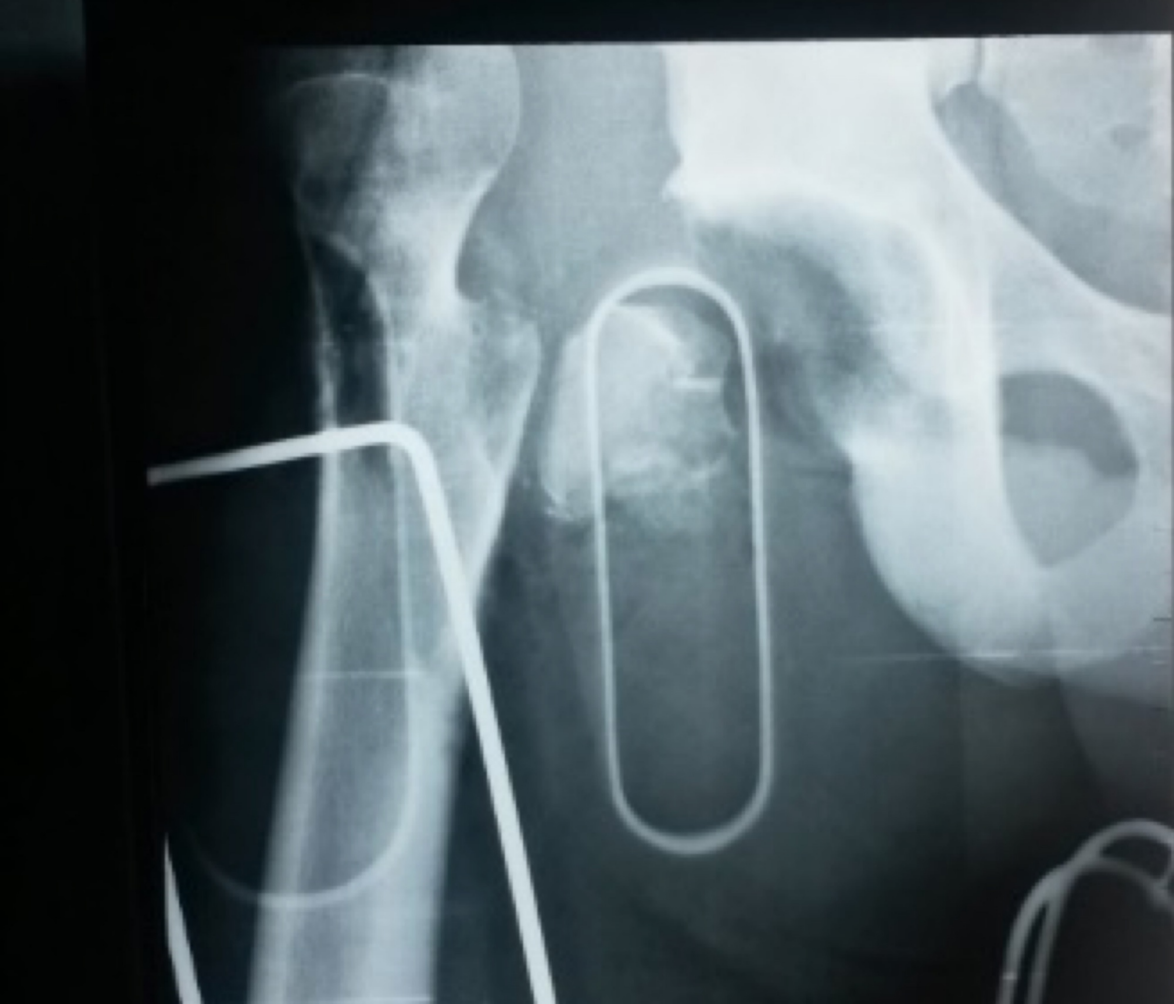
A radiograph of the right hip showing the dislocation with a fracture of the greater trochanter

After a clinical evaluation and imaging tests that excluded cranial or abdominal disorders, we prioritized the neurovascular examination of the affected limb, which did not present complications, and the protection of the femoral head with the use of moistened gauze and saline solution. Radiographs in the anteroposterior view of the right hip showed a hip dislocation with a greater trochanter fracture (Figure 2).

An exhaustive irrigation of the acetabular cavity and the exposed femur was performed, using 10 liters of saline solution at 9% when the patient was in the surgical room. The procedure happened under sedation and spinal anesthesia. A large debridement of muscle, fascia, and bone tissues was required to remove all the devitalized tissue, considered viable only when active bleeding and the clean appearance of the open wound was observed through direct vision by the surgeons.

**Figure 3:**
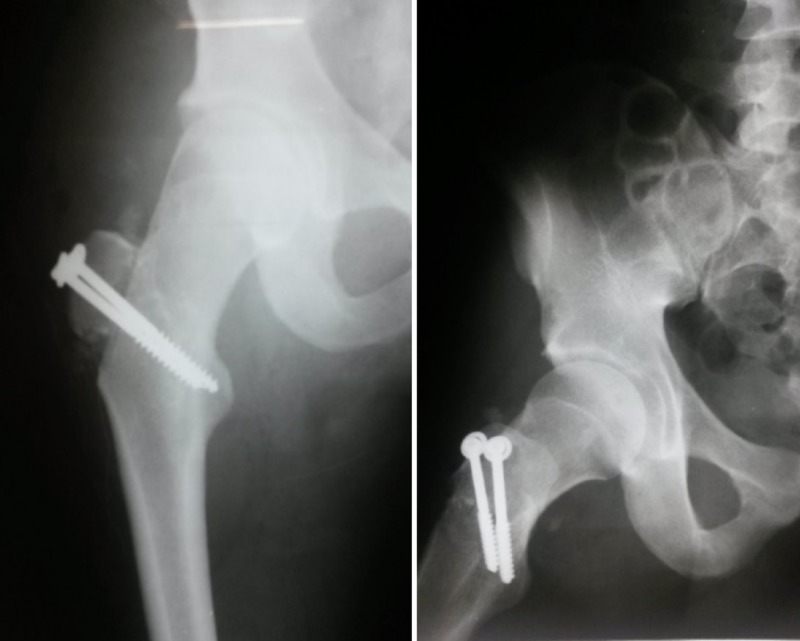
Image of a radiograph after surgery, showing hip reduction and the fixation of the greater trochanter

**Figure 4:**
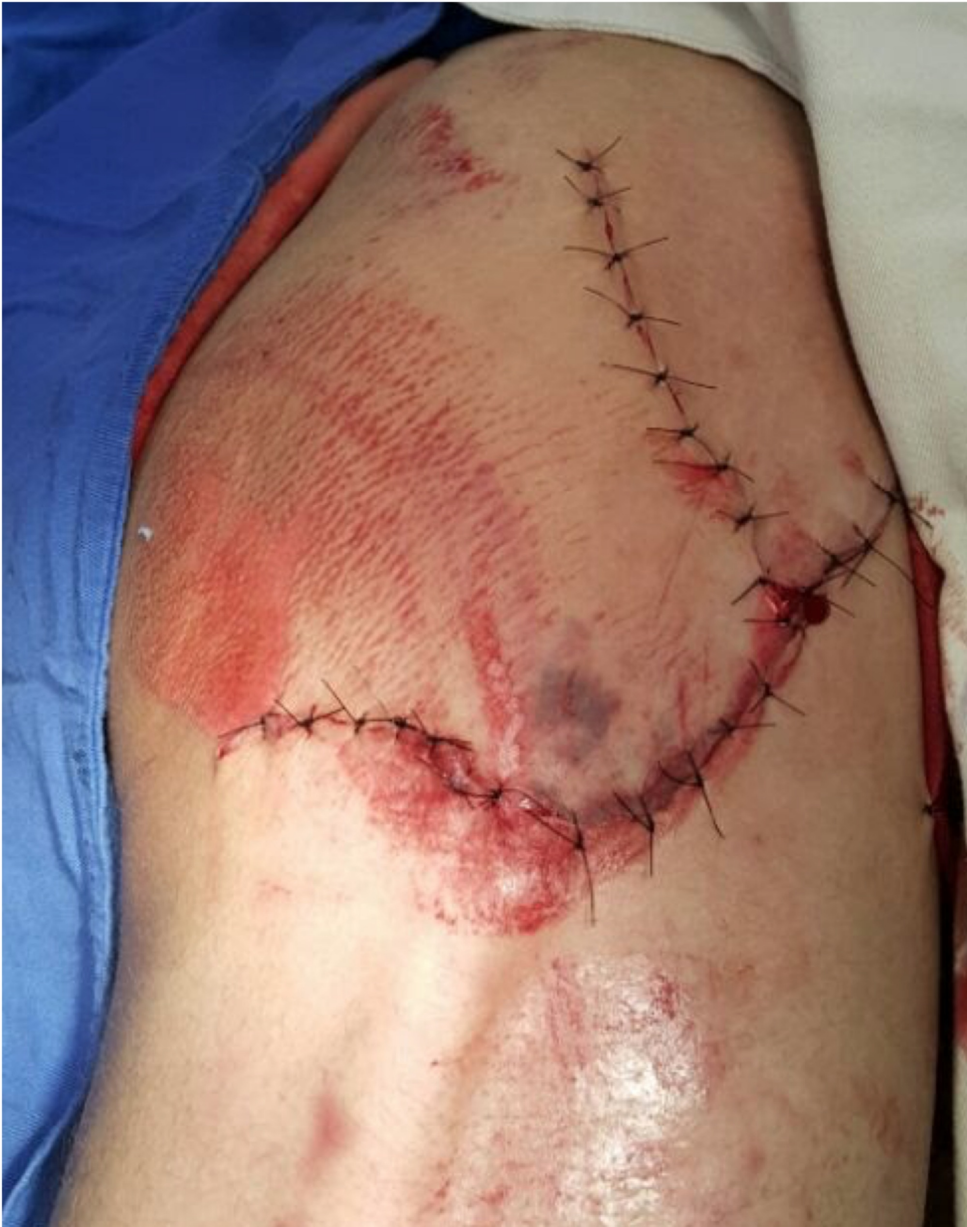
Picture of the lesion, showing the closure of the wound after surgery

The fractured fragment of the greater trochanter was fixed with two 6.5 mm cancellous screws and washers at the proximal end of the femur (Figure 3). After a revision of the debridement sites and radioscopic control of the hip reduction and fixation, the wound was closed (Figure 4).

**Figure 5:**
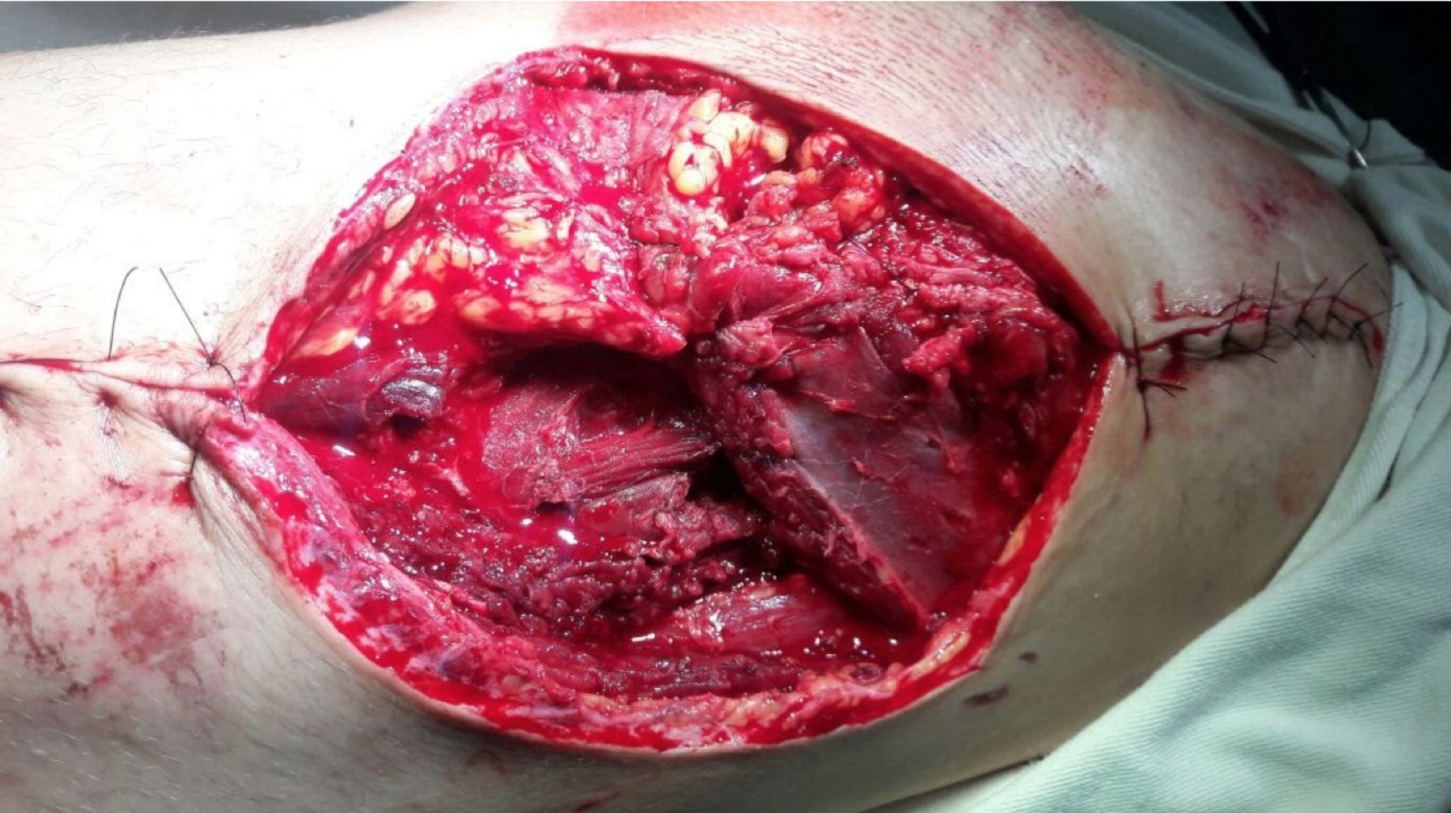
Picture of the wound after second-look surgery

**Figure 6:**
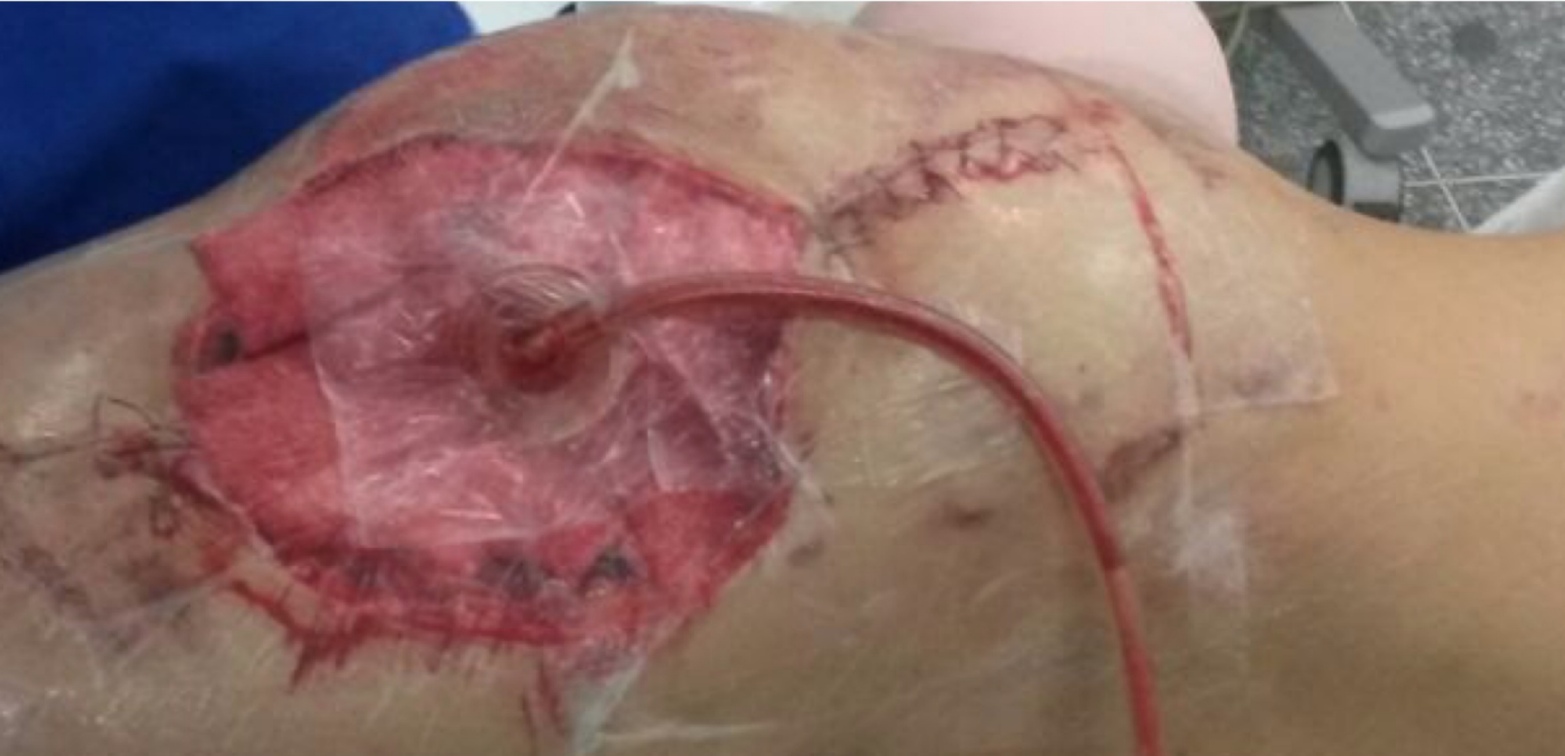
Picture of the vacuum-assisted closure of the wound

After the first 48 hours of surgery, the wound was releasing a significant amount of secretion, bloody and serum like, and a strong odor was observed, with no laboratory exams indicating infection. At this time, a new surgical procedure (second look) with greater aggressiveness was obtained, removing all devitalized tissue and bad-in-appearance cutaneous cover, which was not necrotic but had an unhealthy appearance (Figure 5). A vacuum-assisted closure was used (Figure 6).

**Figure 7:**
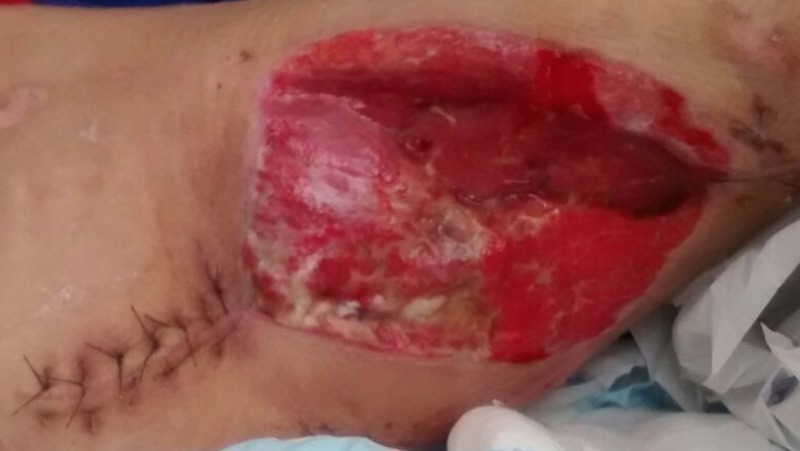
Picture of the wound after four weeks of vacuum-assisted closure

The vacuum-assisted closure was changed every week for four weeks until the appearance of granulation tissue at the surface of the surgical wound (Figure 7). During this period, the patient's laboratory exams showed a drop in the hemoglobin level, resulting in a 7.1 g/dl result, which was corrected with a transfusion of 600 ml of red blood cell (RBC) concentrate. As a rehabilitation procedure, we started daily physiotherapy with passive limb mobility and activity, within pain limits, with no load on the right hip.

In the fifth week, a new surgical procedure was performed for skin grafting. It was performed with no major occurrences and implantation of the graft was successful. Shortly after the removal of the stitches from the graft surgery, in about eight weeks, partial weight bearing on the affected limb was initiated with the use of two crutches.

**Figure 8:**
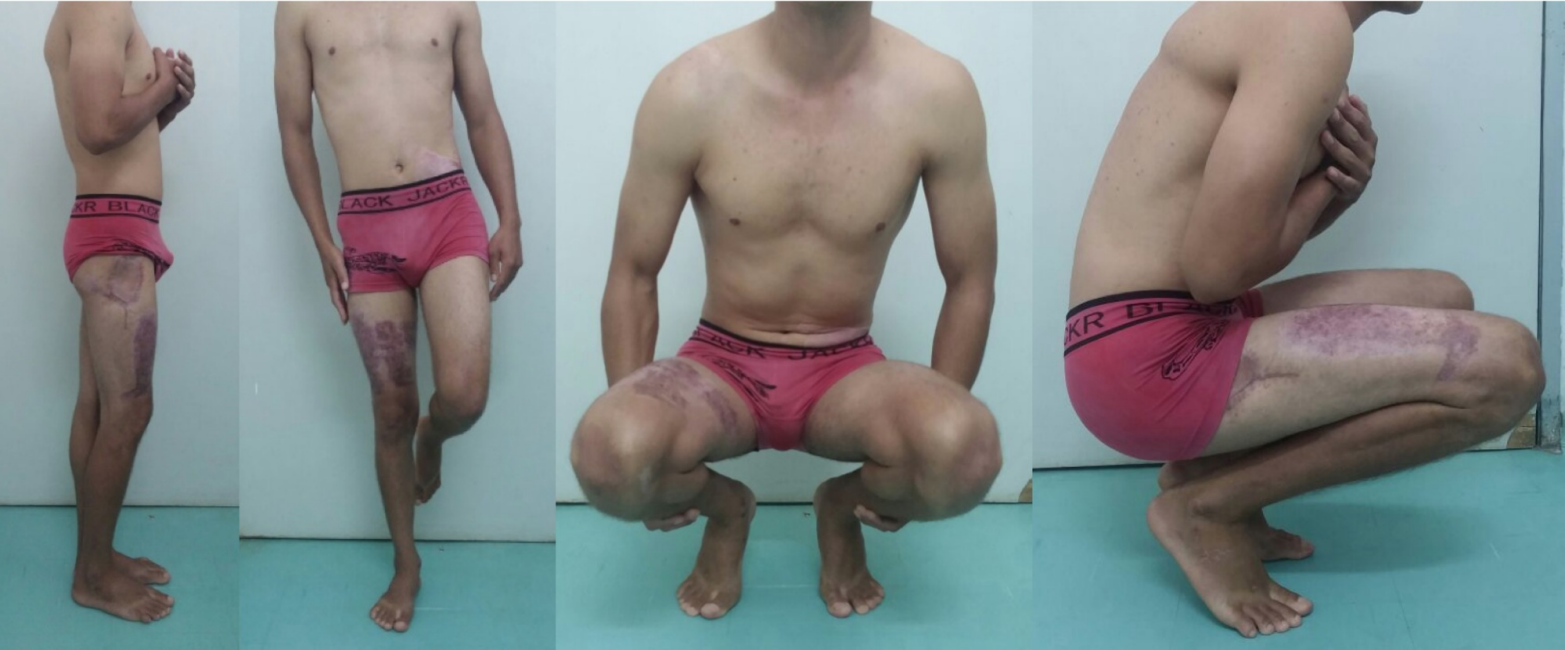
Pictures of the patient at the one-year follow-up

**Figure 9:**
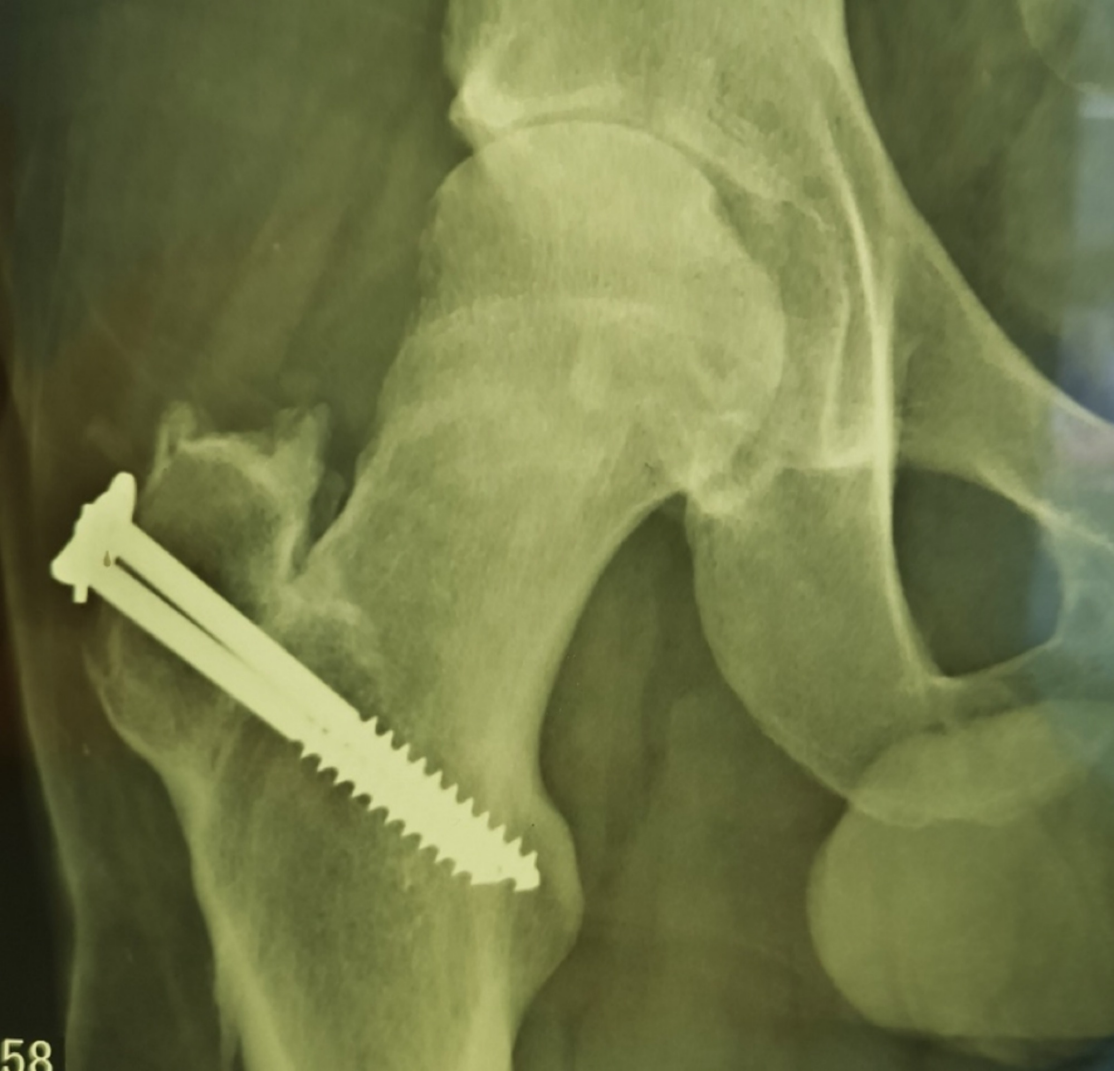
Radiograph showing the healing of the greater trochanter fracture and a narrowing of the hip joint space

**Figure 10:**
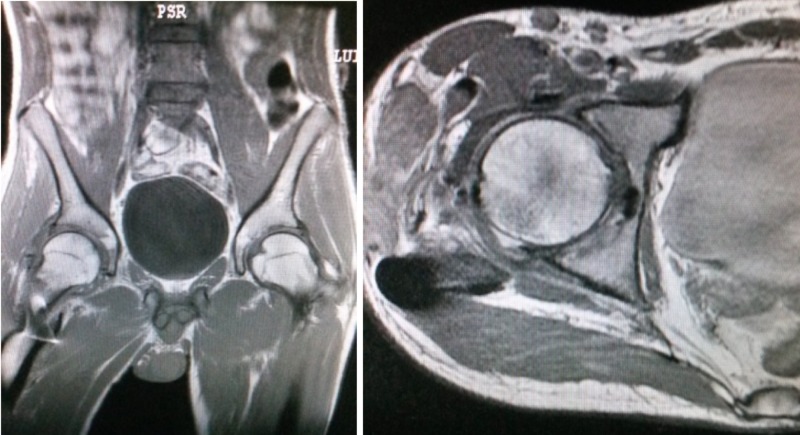
Pictures of the MRI, showing the reduction of the articular space and no necrosis of the femoral head

After a one-year follow-up, the patient had good mobility of the affected limb (Figure 8) without significant pain during mobilization and examination of the joint, with a Harris Hip Score of 93 points. The radiograph showed a decrease of the articular space in the right hip (Figure 9) but the magnetic resonance imaging (MRI) showed no necrosis of the femoral head. (Figure 10). He was able to ride a bicycle, run, and do squats. 

## Discussion

An open anterior dislocation of the hip is a severe trauma with only a few reports described in the literature. Therefore, due to the rarity of the cases, especially in adult patients, there is a lack of treatment guidelines [1].

An early diagnosis with image evaluation is very important for initial treatment and follow-up [2].

Immediate care with precautions taken during the reduction and subsequent debridement procedures, followed by the use of vacuum-assisted wound closure, may be a determinant of good outcomes by avoiding the occurrence of a deep infection [3].

Load restriction and early rehabilitation may have been a differential to avoid the complications of femoral head cartilage lesions, as greater pain limits the range of movement [4-5].

As seen earlier in literature, the fracture of the greater trochanter could be a protective factor for femoral head vascularization, especially if the fragment suffers a posterior deviation, sometimes being able to preserve the insertion of the external rotators. This may have been a reason for the maintenance of the medial circumflex artery close to the greater trochanter, allowing vascular restoration on the femoral head after reduction and fixation [6].

There is still no protocol or guideline for this type of fracture. Therefore, it is very clear that taking precautions during procedures such as reduction, cleaning, fixation, and rehabilitation is very important to achieve a better result and avoid or postpone the complications of this very serious fracture.

## Conclusions

The very low number of cases like this in the literature leads us to affirm that there is no specific protocol to be followed. A functional limitation of the hip was imposed according to what was described previously in the literature. However, the absence of osteonecrosis, until this moment, showed a different result from all the case reports analyzed by the authors. The maintenance of vascularization from the femoral head made us believe that the association of a greater trochanter fracture with this pathology may be the reason for this outcome.
