# Correction: Traumatic Open Anterior Hip Dislocation in an Adult Male: A Case Report

**DOI:** 10.7759/cureus.c16

**Published:** 2019-01-04

**Authors:** Anderson Freitas, Vincenzo Giordano Neto, Patrick F Godinho, Silvio L Macedo, Vinicius F Ribeiro de Oliveira, Gary Alan A Montano, Celio Silva, Vanessa C Bandeira

**Affiliations:** 1 Instituto De Pesquisa E Ensino-Ipe, Hospital Ortopedico E Medicina Especializada - Home, Brasilia, BRA; 2 Orthopedics, Hospital Municipal Miguel Couto, RIo De Janeiro, BRA; 3 Instituto De Pesquisa E Ensino-Ipe, Hospital Ortopedico E Medicina Especializada-Home, Brasilia, BRA; 4 Instituto De Pesquisa E Ensino-Ipe, Hospital Ortopedico E Medicina Especializada - Home, Braslia, BRA

A short video embed showing the patient riding a bike, running, and squatting has been removed from the article as the video was removed from YouTube.

